# Quantitative assessment of intrapulmonary vessel volume by CTPA in vasculitis patients with pulmonary vascular involvement

**DOI:** 10.1186/s12880-026-02354-8

**Published:** 2026-04-20

**Authors:** Ying Ming, Sirong Piao, Ruijie Zhao, Jiaru Wang, Zicheng Liao, Lan Song, Ran Xiao, Rui Zhao, Zhuangfei Ma, Bing Li, Fuling Zheng, Wei Song

**Affiliations:** 1 Department of Radiology, Peking Union Medical College Hospital, Chinese Academy of Medical Sciences and Peking Union Medical College, Beijing, 100730 China; 2 Canon Medical Systems (China), No.3, Xinyuan South Road, Chaoyang District, Beijing, 100027 China

**Keywords:** Computed tomography, Vasculitis, Three-dimensional

## Abstract

**Purpose:**

To compare intrapulmonary vessel volume (IPVV) on computed tomography pulmonary angiography (CTPA) between vasculitis patients with pulmonary vascular involvement and CTPA-negative subjects.

**Methods:**

This study included 207 vasculitis patients with pulmonary vascular involvement between March 2019 and November 2024 and 202 CTPA-negative subjects between February 2019 and February 2025. A computer-aided pulmonary vascular segmentation algorithm was employed to automatically measure total intrapulmonary vessel volume (TIPVV), intrapulmonary arterial vessel volume (IPVVa) and intrapulmonary venous vessel volume (IPVVv). Additionally, IPVVs were analyzed and compared within five specific vessel diameter groups: 0.8–1.6 mm, 1.6–2.4 mm, 2.4–3.2 mm, 3.2–4.0 mm, and > 4.0 mm.

**Results:**

TIPVV and IPVVv showed no significant differences between groups. The IPVVa measured in CTPA-negative subjects was 47.79 (42.48, 54.10) mL·m^− 2^, while that in vasculitis patients with pulmonary vascular involvement was 44.86 (39.33, 52.58) mL·m^− 2^. The IPVVa in vasculitis patients with pulmonary vascular involvement was significantly lower than that in CTPA-negative subjects (*p* < 0.01). In pulmonary arteries with diameters of 0.8–1.6 mm and 2.4–3.2 mm, the IPVVa in vasculitis patients with pulmonary vascular involvement was lower than that in CTPA-negative subjects (*p* < 0.05). In pulmonary veins with diameters of 1.6–2.4 mm and 3.2–4.0 mm, the IPVVv in vasculitis patients with pulmonary vascular involvement was higher than that in CTPA-negative subjects (*p* < 0.05).

**Conclusions:**

The computer-aided pulmonary vascular segmentation algorithm can automatically measure IPVV, enabling quantitative assessment of small pulmonary vessel involvement in vasculitis.

## Introduction

Vasculitis is characterized by idiopathic inflammation of blood vessel walls, affecting blood vessels of various sizes. It primarily includes large-vessel vasculitis, medium-vessel vasculitis, small-vessel vasculitis, variable vessel vasculitis, single-organ vasculitis, vasculitis associated with systemic disease, and vasculitis associated with probable etiology [1, 2]. Several studies have indicated that Takayasu arteritis and giant cell arteritis (GCA) primarily involve the pulmonary large vessels [3], while antineutrophil cytoplasmic antibody (ANCA)-associated vasculitis (AAV) predominantly affects the pulmonary medium and small vessels [4].

Autopsy reports reveal that pulmonary artery involvement in Takayasu arteritis occurs in 20%–56% of cases. Additionally, imaging studies indicate pulmonary artery involvement rates of 33%–86% in patients with Takayasu arteritis [5]. Approximately 50% of these patients develop significant pulmonary hypertension, primarily due to pulmonary artery stenosis or occlusion [6]. The absence of systemic vascular involvement often leads to misdiagnosis or delayed diagnosis in patients with vasculitis with pulmonary artery involvement [7, 8]. Consequently, pulmonary hypertension is frequently detected at an advanced stage, contributing to increased hospital readmissions and higher cardiac mortality [9].

Although pulmonary arterial pressure measurements and pulmonary angiography can be used to directly evaluate abnormalities in specific regions of the pulmonary vasculature, these procedures require invasive cardiac catheterization in routine clinical practice [10]. Computed tomography pulmonary angiography (CTPA) has emerged as the preferred imaging modality for evaluating pulmonary vasculitis, given its non-invasive nature and practical feasibility. Visual assessment and quantitative analysis using CTPA of pulmonary vascular abnormalities, such as vessel dilation, stenosis and pruning, have gained considerable interest among researchers.

Prior studies have linked the cross-sectional area percentage of small pulmonary vessels (%CSA) with pulmonary vascular changes, identifying %CSA < 5 mm^2^ as an independent predictor of pulmonary hypertension [11]. However, %CSA is measured based on two-dimensional slices and thus cannot accurately reflect the overall pulmonary vasculature. Intrapulmonary vessel volumes (IPVV) can be non-invasively extracted, analyzed, and quantified, providing valuable insights into various lung diseases [12–15]. The computer-aided pulmonary vascular segmentation algorithm allows for precise identification and detailed morphometric analysis of pulmonary arteries and veins, enabling labeling with high accuracy and minimal manual intervention.

This study employed a computer-aided pulmonary vascular segmentation algorithm to analyze abnormalities in small pulmonary vessels in vasculitis patients with pulmonary vascular involvement. By comparing IPVV between groups, this study aimed to assess the algorithm’s potential in identifying small pulmonary vessel abnormalities.

## Methods

### Patients

This retrospective study was approved by the Institutional Review Board of our Hospital (No. I-23PJ2181) and exempted from informed consent. Between March 2019 and November 2024, 261 patients diagnosed with vasculitis and clinically suspected of pulmonary vascular involvement underwent CTPA. Pulmonary vascular involvement was defined as luminal stenosis, occlusion, or wall thickening of pulmonary arteries identifiable on CTPA. In cases of significant proximal stenosis or occlusion, associated distal vascular pruning was also considered supportive evidence of vascular involvement. Images were independently reviewed by two radiologists with 4 and 5 years of experience in chest CT interpretation. Discrepancies were resolved by a senior radiologist with 25 years of experience to confirm pulmonary vascular abnormalities. Among the 261 patients, 252 patients showed pulmonary vascular abnormalities on CTPA. After applying the inclusion and exclusion criteria, 207 patients were ultimately included in the study.

The inclusion criteria were as follows: (1) clear images with accurate segmentation of lung vessels; (2) a minimum average CT value of 250 Hounsfield Units (HU) to ensure adequacy, with optimal pulmonary artery opacification achieved with an enhancement level between 250 and 300 HU [16, 17]; (3) confirmation that patients met the 2022 clinical diagnostic criteria for vasculitis established jointly by the American College of Rheumatology (ACR) and the European Alliance of Associations for Rheumatology (EULAR) [18–20].

The exclusion criteria were as follows: (1) history of thoracic surgery; (2) presence of lung cancer or other space-occupying lesions, chronic obstructive pulmonary disease (COPD); (3) thoracic deformity; (4) inadequate vascular enhancement and poor image quality with severe artifacts that could not be processed by the algorithm; (5) significant imaging abnormalities, such as infectious pneumonia, consolidations, severe emphysema, atelectasis and interstitial lung disease that could affect lung analysis; (6) pulmonary arteriovenous malformations or Behcet’s disease with aneurysmal dilatation of the pulmonary vasculature; (7) incomplete data sets.

Patients with malignant tumors, COPD, pulmonary vascular malformations, vasculitis, connective tissue disease, idiopathic pulmonary arterial hypertension and pulmonary embolism were excluded. Patients with a history of thoracic surgery or thoracic deformity were also excluded. A total of 205 subjects who underwent CTPA between February 2019 and February 2025 for symptoms such as dyspnea or chest pain and had negative CTPA results were initially considered. The CTPA images were independently reviewed by two radiologists. Discrepancies were resolved by a senior radiologist to confirm the absence of pulmonary vascular abnormalities. According to the inclusion and exclusion criteria, 202 subjects were included in the final analysis.

The inclusion criteria were as follows: (1) clear images suitable for accurate segmentation; (2) average CT value ≥ 250 HU to ensure adequate image quality.

The exclusion criteria were as follows: (1) inadequate vascular enhancement and poor image quality with severe artifacts that could not be processed by the algorithm; (2) incomplete data sets.

**Fig. 1:**
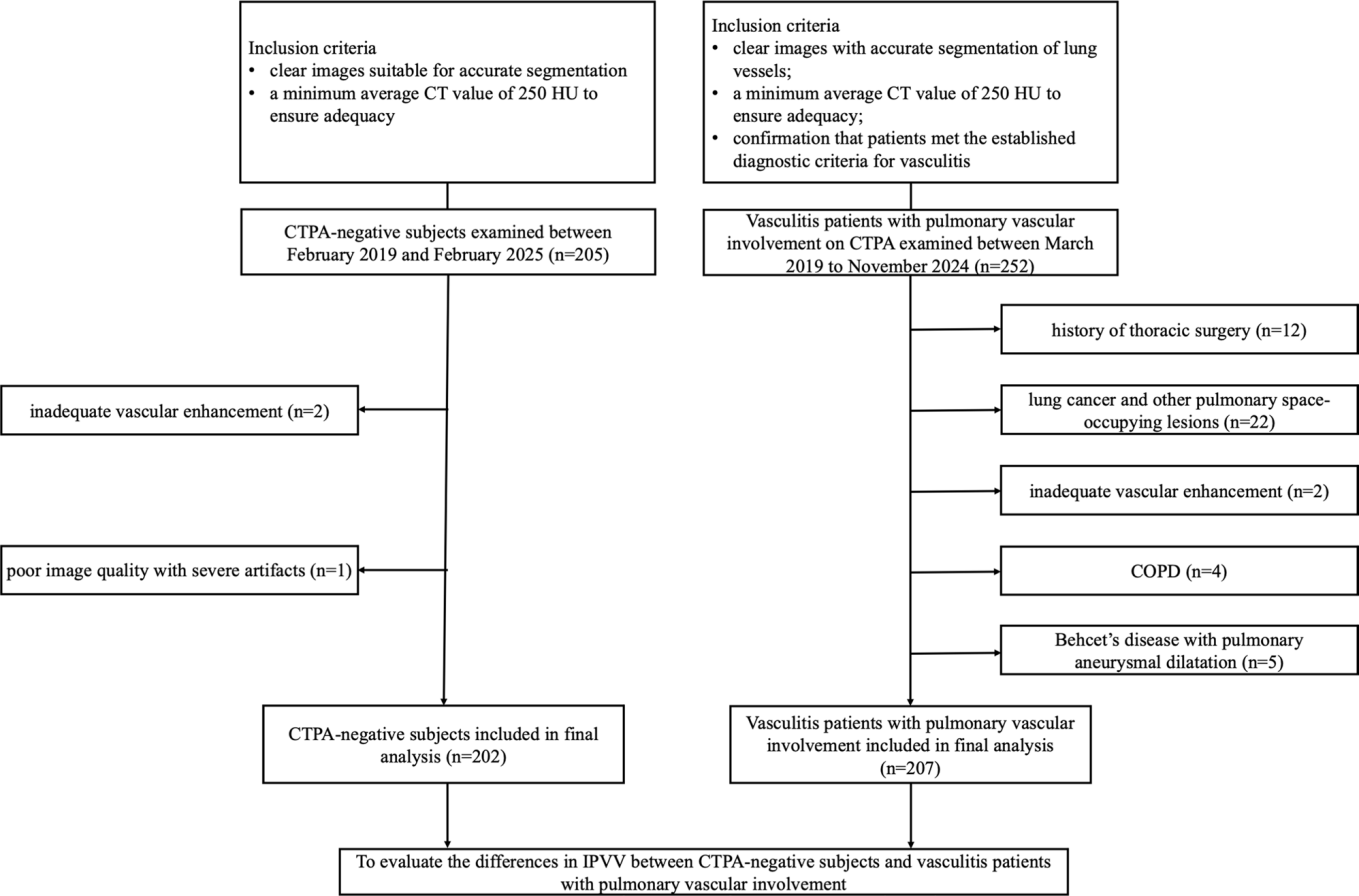
Flowchart depicting the study selection process

The demographic information of the cohort is summarized in Table 1. The study flowchart is shown in Fig. 1.

**Table 1:** Demographic information of the cohort

	CTPA-negative subjects	Vasculitis patients	*P* value
Number of subjects	202	207	-
age, years (median, IQR)	45 (32, 60)	34 (27, 44)	< 0.001
gender			< 0.001
male	74 (37%)	30 (14%)	
female	128 (63%)	177 (86%)	
height, cm (median, IQR)	164 (160, 173)	163 (160, 168)	0.008
weight, kg (median, IQR)	66 (58, 75)	59 (52, 65)	< 0.001
BSA (average), m²(median, IQR)	1.72 (1.61, 1.85)	1.61 (1.54, 1.72)	< 0.001

Categorical data expressed as n (number) and %. IQR, Interquartile Range; BSA, body surface area

### CT Image acquisition

All CTPA images were acquired using three different CT systems: Somatom Definition Flash or Somatom Force (Siemens Healthineers, Forchheim, Germany), Philips IQon CT (Philips Medical Systems, Best, Netherlands) and Aquilion ONE GENESIS (Canon Medical Systems, Tokyo, Japan). Before imaging, all participants underwent breath-holding training. During the scan, individuals were positioned supinely, with hands cradling their heads. A whole-lung scan from the pulmonary apex to the base was obtained during a breath-hold at the end of deep inhalation. Contrast-enhanced imaging was performed by administering 40 mL of iodinated contrast (Iopromide, Ultravist 370, Bayer HealthCare) via the antecubital vein at 4 mL/s, followed by 30 mL saline injection at the same rate. The CT scanning parameters included a voltage of 120 kVp, automatic tube-current modulation activated settings, and collimation setting of 64 × 0.6 mm (Somatom Definition Flash, Somatom Force), 64 × 0.625 mm (Philips IQon CT) or 80 × 0.5 mm (Aquilion ONE GENESIS). The rotation time was set at 0.5 s and an image matrix of 512 × 512. All images were reconstructed to a 1-mm slice thickness using a standard algorithm. All resulting image files were saved in Digital Imaging and Communications in Medicine (DICOM) format.

### Quantitative assessment of pulmonary vessels

**Fig. 2:**
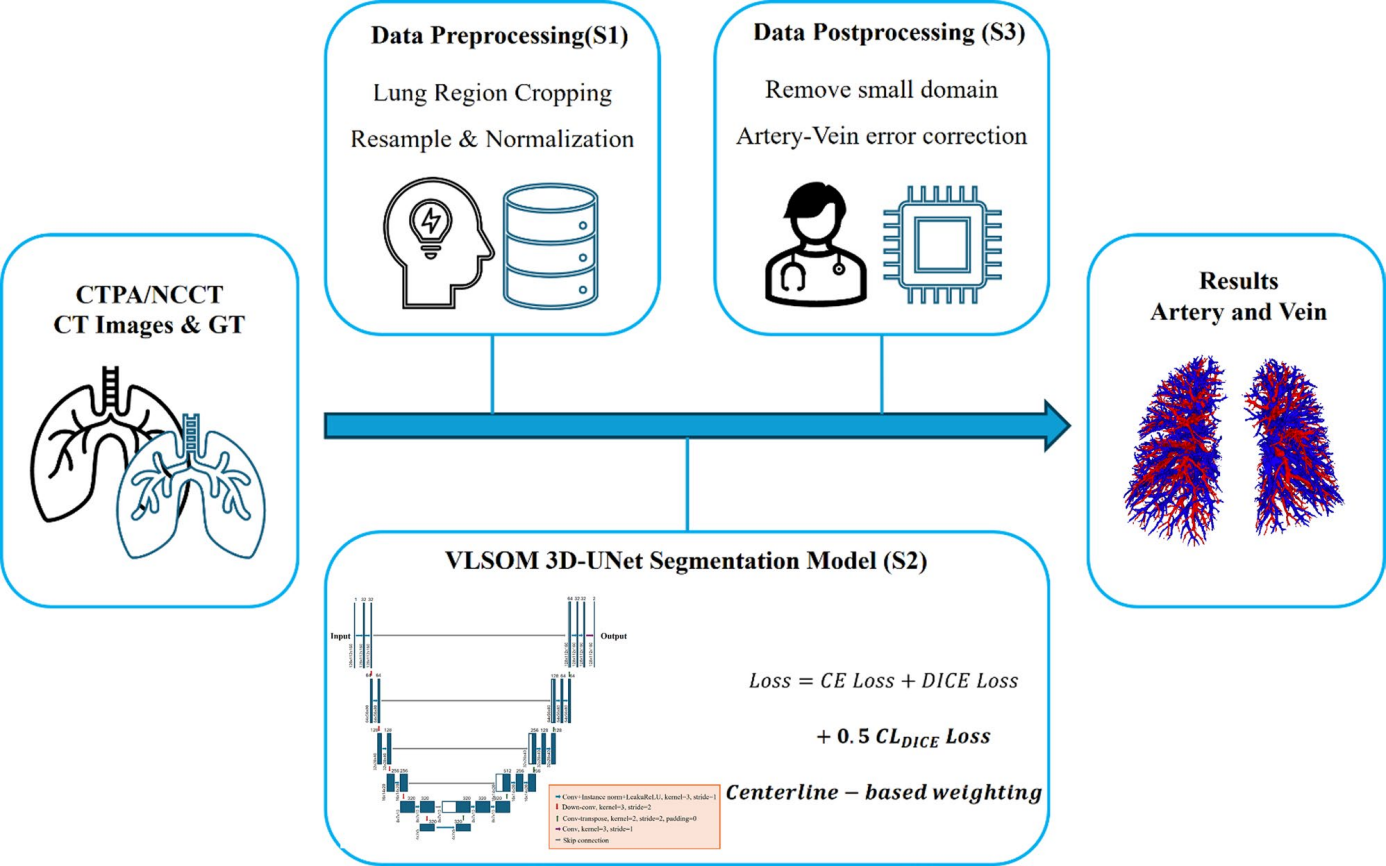
Workflow of the computer-aided pulmonary vascular segmentation algorithm during the development stage

The anonymized datasets were transferred to separate workstations for advanced post-processing. Pulmonary vessels in the CTPA image data were automatically segmented using a pulmonary vascular segmentation algorithm (Abierto Vision post-processing workstation, Canon Medical Systems). The algorithm applied a 3D U-Net deep learning model with a topology-preserving loss function for pulmonary vessel segmentation and artery–vein classification [21]. The workflow of the computer-aided pulmonary vascular segmentation algorithm during the development stage is illustrated in Fig. 2. The model was trained using high-quality annotated CT datasets from multiple vendors and international centers. Quantitative validation on an independent testing set demonstrated high segmentation performance for CTPA data, achieving Cl-DICE, Cl-Recall, and Recall values of 0.892, 0.861, and 0.924, respectively, as previously reported in our methodological preprint [21]. Based on the segmentation results, the IPVV was automatically calculated.

**Fig. 3:**
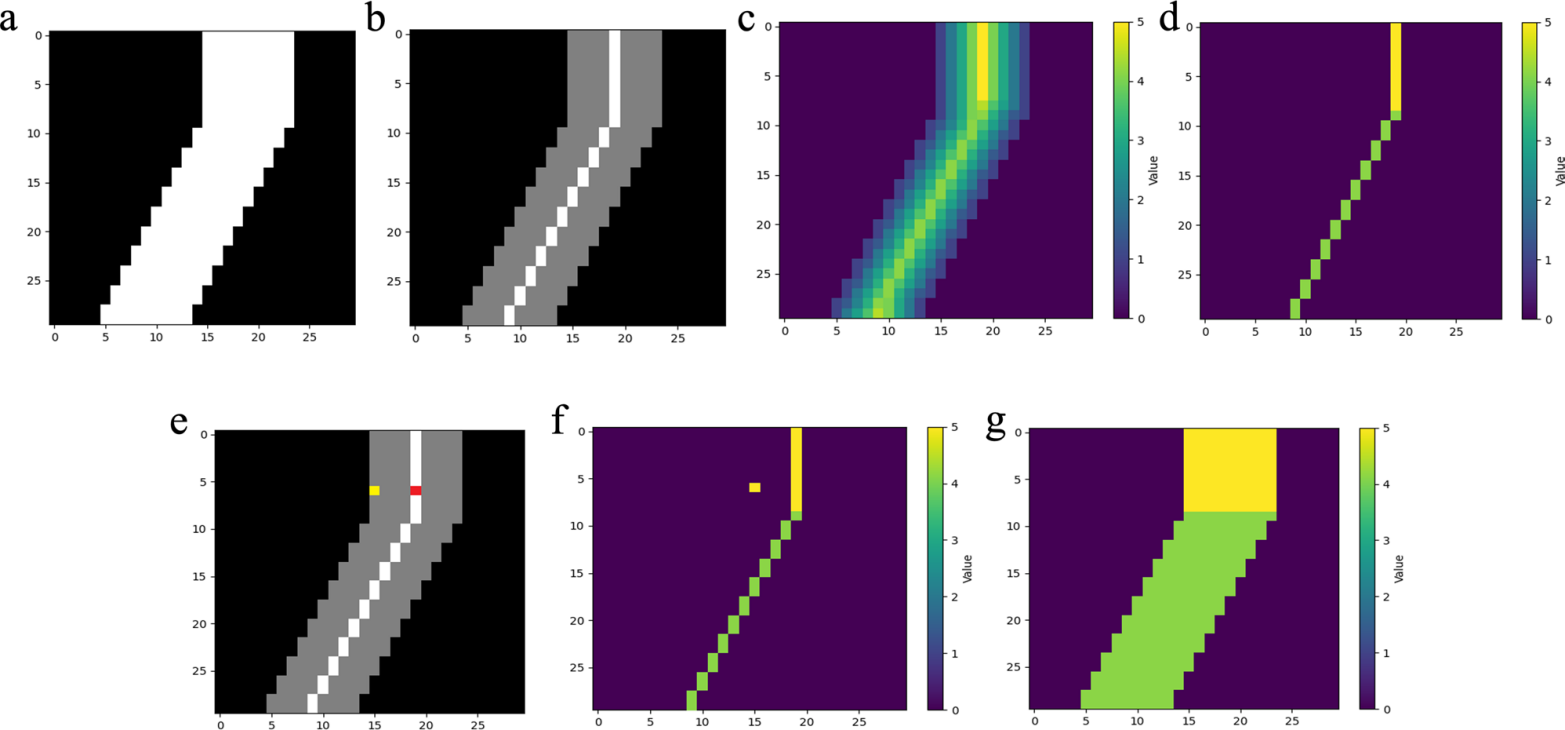
Computational workflow for vascular radius estimation. (**a**) Phantom vessel mask: binary mask of the phantom vessel. (**b**) Centerline extraction: extraction of the vessel centerline. (**c**) Distance map: Euclidean distance transform (EDT) computed from the vessel boundary to the interior. (**d**) Centerline radius values: extraction of radius values at the vessel centerline by mapping the EDT onto the centerline mask. (**e**) Nearest neighbor search: for each non-centerline voxel (yellow), the algorithm identifies the nearest centerline point (red) within the Euclidean space. (**f**) Distance value assignment: assigns the corresponding radius value of the centerline voxel to the target non-centerline voxel. (**g**) Radius propagation: each voxel within the vessel mask is assigned the radius value of its corresponding nearest centerline voxel

Besides, for radius-level analysis, we introduced a vascular radius calculation algorithm that converts binary vessel masks into spatially continuous radius distributions: (1) Centerline & Distance Map Extraction: skeletonized centerlines are extracted, and a Euclidean distance transform quantifies minimal boundary distances for all foreground voxels. (2) Radius Assignment: the distance value at the centerline position inherently represents the cross-sectional radius of corresponding vascular lumen. Thus, the centerline-to-boundary distance equals the vessel radius for each centerline position. (3) Hierarchical Radius Propagation: for each non-centerline foreground target voxel, candidate centerline points are first assigned through a spatial constraint filter – only if the distance between non-centerline voxel and centerline voxel is smaller than corresponding radius. Subsequently, a nearest neighbor assignment selects the geometrically closest candidate centerline point, with its corresponding radius value assigned to the target non-centerline voxel. This hierarchical matching ensures continuous radius propagation throughout the vascular tree while maintaining physiological plausibility. Figure 3 shows a computational workflow for vascular radius estimation.

The segmented 3D pulmonary vessels were rendered using 3D Slicer software (version 5.6.1). A computer-aided pulmonary vascular segmentation algorithm was applied to automatically label intrapulmonary arteries and veins, with arteries marked in red and veins in blue. The segmentation results were visually assessed and independently reviewed by two experienced chest radiologists, blinded to the clinical data. When necessary, minor manual refinements were performed to ensure anatomical plausibility and correct vessel classification. These refinements were infrequent and primarily limited to artery-vein crossing regions or small-caliber vessel segments. In cases of disagreement, a third senior radiologist with 25 years of experience in chest CT interpretation reviewed the segmentation and finalized the corrections by consensus. Corrections mainly involved boundary adjustment or local reclassification and did not require large-scale modification of the vascular tree. Final quantitative measurements were derived from the quality-controlled segmentations. Based on the vessel segmentation results, the total intrapulmonary vessel volume (TIPVV), intrapulmonary arterial vessel volume (IPVVa) and intrapulmonary venous vessel volume (IPVVv) were calculated for the vasculitis patients with pulmonary vascular involvement and CTPA-negative subjects.

A previous study [22] categorized small pulmonary vessels based on diameter thresholds of < 0.8 mm, < 1.2 mm and < 1.6 mm, and found that in patients with severe pulmonary hypertension, the IPVV of vessels with diameters < 1.6 mm was more strongly associated with survival prediction. Due to the resolution limitations of CT imaging, vessels with diameters < 0.8 mm could not be reliably used for prognostic assessment. Moreover, injury to the distal small pulmonary vessels has a minimal impact on TIPVV, and larger pulmonary vessels better reflect overall pulmonary vascular health. Based on these findings, the present study optimized vessel size categorization by setting 0.8 mm as the minimum diameter threshold, and further subdivided vessels into five groups: 0.8–1.6 mm, 1.6–2.4 mm, 2.4–3.2 mm, 3.2–4.0 mm and > 4.0 mm. For each group, the IPVVa and IPVVv were measured separately.

Given that vessels with diameters between 0.8 mm and 3.2 mm anatomically correspond to subsegmental pulmonary vessels and their distal branches [11], further subdividing this range for color coding would result in visually indistinct and mottled patterns that are difficult to differentiate by the naked eye. Therefore, vessels with diameters of 0.8–3.2 mm were coded in red, vessels with diameters of 3.2–4.0 mm were coded in yellow, and those with diameters of > 4.0 mm in blue.

The TIPVV was defined as the combined volume of IPVVa and IPVVv, excluding those at the lung hilum. According to previous literature [22], the walls of intrapulmonary arteries and veins are extremely thin, the IPVV includes vessel walls and luminal blood. As pulmonary artery size is associated with body surface area (BSA) [23], the DuBois formula [24] was used to calculate BSA for each subject based on their height and weight. Pulmonary vascular parameters were then corrected and normalized accordingly:

$$\:BSA\left({m}^{2}\right)={Weight\left(kg\right)}^{0.425}\times\:{Height\left(cm\right)}^{0.725}\times\:0.007184$$

### Statistical analysis

All statistical analyses were performed using SPSS (version 26; IBM, New York, USA), while GraphPad Prism (version 9.5.1; GraphPad Software, San Diego, USA) was used for data visualization. Continuous variables with normal distribution are presented as mean and standard deviations, while the non-normally distributed variables are presented as medians and interquartile ranges (first and third quartiles). The categorical variables are reported as frequency (percentage). For continuous data with normal distribution, Student’s t-test was employed to compare the IPVV between vasculitis patients with pulmonary vascular involvement and CTPA-negative subjects. Non-normally distributed continuous data were analyzed using the Mann-Whitney U test for comparison. A multiple linear regression model with robust standard errors was performed to evaluate the independent association between group and IPVVa, adjusting for age, sex, and BSA. A p-value < 0.05 was considered statistically significant.

## Results

### Study participants

According to the inclusion and exclusion criteria, 45 vasculitis patients with pulmonary vascular involvement were excluded: 12 due to thoracic surgery, 9 with lung cancer or other space-occupying lesions, 4 with COPD, 13 with significant imaging abnormalities, 5 with pulmonary aneurysmal dilatation associated with Behcet’s disease and 2 with inadequate vascular enhancement. Ultimately, 207 patients were included, with a median age of 34 (13–67) years. Of these, 30 (14%) were male and 177 (86%) were female.

According to the inclusion and exclusion criteria, 3 CTPA-negative subjects were excluded: 1 with severe artifacts and 2 with inadequate vascular enhancement. A total of 202 CTPA-negative subjects were included in the study, with a median age of 45 (11–83) years; of these, 37% were male and 63% were female.

### IPVV of CTPA-negative subjects and vasculitis patients with pulmonary vascular involvement

A comparison of TIPVV, IPVVa and IPVVv between CTPA-negative subjects and vasculitis patients with pulmonary vascular involvement is presented in Table 2. The IPVVa in vasculitis patients with pulmonary vascular involvement was significantly lower than that in CTPA-negative subjects (*p* < 0.01). No statistically significant differences were found in TIPVV or IPVVv between the two groups (*p* > 0.05). After adjustment for age, sex, and BSA, group remained significantly associated with IPVVa (*p* < 0.001). Sex was also independently associated with IPVVa (*p* = 0.042), whereas age and BSA were not statistically significant. These findings suggest that the reduced IPVVa observed in vasculitis patients is unlikely to be fully explained by baseline demographic differences.

**Table 2:** Comparison of IPVVs between CTPA-negative subjects and vasculitis patients with pulmonary vascular involvement

IPVV	CTPA-negative subjects	Vasculitis patients	Z	*P*
TIPVV, mL·m^− 2^	104.10 (94.97, 116.44)	104.00 (91.32, 118.59)	-0.644	0.519
IPVVa, mL·m^− 2^	47.79 (42.48, 54.10)	44.86 (39.33, 52.58)	-2.914	0.004
IPVVv, mL·m^− 2^	57.03 (51.07, 63.13)	58.15 (51.86, 66.49)	-1.415	0.157

Data not conforming to normal distributions are expressed as median (lower quartile, upper quartile). IPVV, intrapulmonary vessel volume; TIPVV, total intrapulmonary vessel volume; IPVVa, intrapulmonary arterial vessel volume; IPVVv, intrapulmonary venous vessel volume

### IPVV of different pulmonary vessel diameter groups in CTPA-negative subjects and vasculitis patients with pulmonary vascular involvement

In pulmonary arteries with diameters of 0.8–1.6 mm and 2.4–3.2 mm, the IPVVa in vasculitis patients with pulmonary vascular involvement was lower than that in CTPA-negative subjects, with a statistically significant difference (*p* < 0.05). In pulmonary arteries with diameters of 1.6–2.4 mm and > 4.0 mm, although vasculitis patients with pulmonary vascular involvement showed slightly lower IPVVa than CTPA-negative subjects, the differences were not statistically significant (*p* > 0.05). In pulmonary arteries with diameters of 3.2–4.0 mm, the IPVVa was slightly higher in vasculitis patients with pulmonary vascular involvement, but the difference was not statistically significant (*p* > 0.05).

**Fig. 4:**
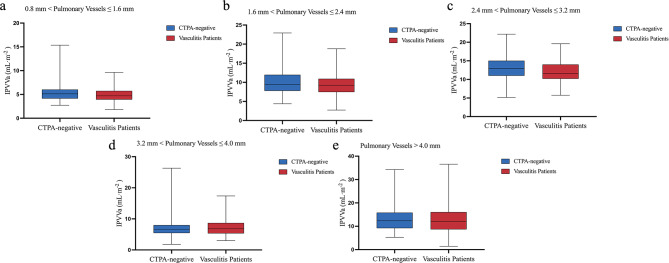
Boxplots of IPVVa across different vessel diameter groups in CTPA-negative subjects and vasculitis patients with pulmonary vascular involvement. IPVVa, intrapulmonary arterial vessel volume

**Fig. 5:**
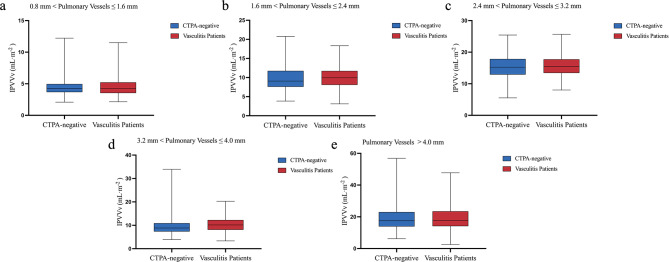
Boxplots of IPVVv across different vessel diameter groups in CTPA-negative subjects and vasculitis patients with pulmonary vascular involvement. IPVVv, intrapulmonary venous vessel volume

The IPVVv in vasculitis patients with pulmonary vascular involvement was greater than in CTPA-negative subjects for all diameter groups. In pulmonary veins with diameters of 1.6–2.4 mm and 3.2–4.0 mm, the IPVVv in vasculitis patients with pulmonary vascular involvement was higher than that in CTPA-negative subjects, with a statistically significant difference (*p* < 0.05), whereas no significant differences were observed in the remaining groups (Table 3) (Figs. 4 and 5).

**Table 3:** Comparison of IPVVs at different vessel diameters between CTPA-negative subjects and vasculitis patients with pulmonary vascular involvement

IPVV	CTPA-negative subjects	Vasculitis patients	Z/t value	*P* value
IPVVa				
0.8 mm < pulmonary vessels ≤ 1.6 mm	5.14 (4.15, 6.03)	4.71 (3.93, 5.75)	-2.367	0.018
1.6 mm < pulmonary vessels ≤ 2.4 mm	9.40 (7.78, 11.97)	9.23 (7.46, 10.93)	-1.602	0.109
2.4 mm < pulmonary vessels ≤ 3.2 mm	12.93 (11.00, 15.03)	11.61 (10.18, 14.03)	-4.069	< 0.001
3.2 mm < pulmonary vessels ≤ 4.0 mm	6.49 (5.44, 7.98)	6.79 (5.31, 8.69)	-0.824	0.410
pulmonary vessels > 4.0 mm	12.34 (9.18, 15.86)	11.95 (8.67, 16.08)	-0.856	0.392
IPVVv				
0.8 mm < pulmonary vessels ≤ 1.6 mm	4.24 (3.67, 4.96)	4.25 (3.53, 5.22)	-0.064	0.949
1.6 mm < pulmonary vessels ≤ 2.4 mm	9.08 (7.58, 11.75)	9.94 (8.08, 11.74)	-2.037	0.042
2.4 mm < pulmonary vessels ≤ 3.2 mm	15.40 ± 3.68	15.67 ± 3.12	-0.778	0.437
3.2 mm < pulmonary vessels ≤ 4.0 mm	8.85 (7.34, 10.89)	10.16 (8.07, 12.27)	-3.736	< 0.001
pulmonary vessels > 4.0 mm	17.52 (13.95, 23.02)	17.76 (14.12, 23.49)	-0.153	0.878

Normally distributed data are expressed as means ± standard deviations, while data not conforming to normal distributions are expressed as median (lower quartile, upper quartile). IPVV, intrapulmonary vessel volume; IPVVa, intrapulmonary arterial vessel volume; IPVVv, intrapulmonary venous vessel volume

### 3D visualization of pulmonary vascular segmentation

**Fig. 6:**
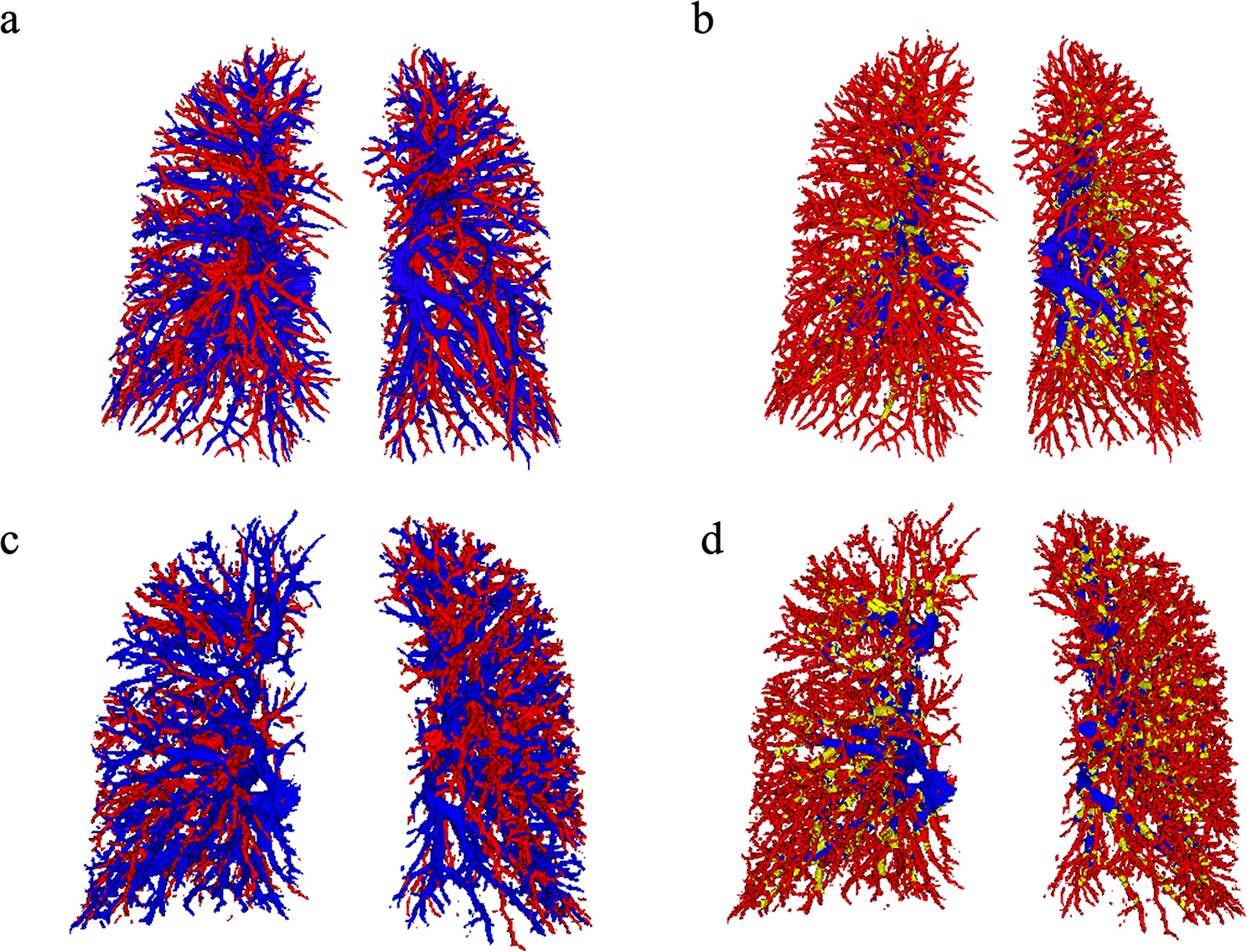
3D visual representation of pulmonary vessel segmentation. (**a**,** b**) Images of a CTPA-negative subject. (**a**) 3D reconstruction of segmented pulmonary arteries (red) and veins (blue). (**b**) Color-coded pulmonary vessels based on diameter: pulmonary vessels 0.8 mm < pulmonary vessels ≤ 3.2 mm are indicated in red, pulmonary vessels 3.2 mm < pulmonary vessels ≤ 4.0 mm are indicated in yellow, pulmonary vessels > 4.0 mm are indicated in blue. (**c**,** d**) Images of a vasculitis patient with pulmonary vascular involvement. (**c**) 3D reconstruction of segmented pulmonary arteries (red) and veins (blue). (**d**) Color-coded pulmonary vessels based on diameter: pulmonary vessels 0.8 mm < pulmonary vessels ≤ 3.2 mm are indicated in red, pulmonary vessels 3.2 mm < pulmonary vessels ≤ 4.0 mm are indicated in yellow, pulmonary vessels > 4.0 mm are indicated in blue. Visual comparison suggests reduced pulmonary arteries and fewer small pulmonary vessels (0.8 mm < pulmonary vessels ≤ 3.2 mm) in vasculitis patients with pulmonary vascular involvement

To visualize pulmonary vascular morphology, 3D images of segmented pulmonary vessels were generated using a computer-aided pulmonary vascular segmentation algorithm, allowing for separate visualization of pulmonary arteries (in red) and pulmonary veins (in blue). The algorithm also enabled segmentation and display of pulmonary vessel segments by pulmonary vessel diameter: 0.8–3.2 mm (red), 3.2–4.0 mm (yellow) and > 4.0 mm (blue). The 3D images demonstrated that vasculitis patients with pulmonary vascular involvement had fewer pulmonary arteries compared to CTPA-negative subjects (Fig. 6a, c), as well as fewer small pulmonary vessels with diameter between 0.8 mm and 3.2 mm (Fig. 6b, d).

## Discussion

Previous studies [11, 25, 26] reported that the %CSA < 5 mm^2^ correlates with pulmonary perfusion, pulmonary hypertension, and emphysema, reflecting small pulmonary vessel remodeling. However, %CSA derived from two-dimensional images cannot fully capture the overall changes in the pulmonary vasculature. Previous research has focused predominantly on the aortic wall thickness but neglected the abnormalities in small distal pulmonary vessels due to technical limitations [27]. In this study, we quantified IPVVs in both CTPA-negative subjects and vasculitis patients with pulmonary vascular involvement using a computer-aided pulmonary vascular segmentation algorithm based on CTPA images, aiming to assess pulmonary vasculature changes and provide insights into small pulmonary vessel abnormalities.

In this study, 86% of the vasculitis patients with pulmonary vascular involvement were female, a proportion significantly higher than that of the CTPA-negative subjects. This disparity may be attributed to the role of estrogen in the development and progression of vasculitis, as well as the presence of numerous immune-related genes on the X chromosome, both of which contribute to a higher risk of autoimmune diseases in females [28–31]. Given that vasculitis is a relatively rare condition and the number of patients with vasculitis at a single center is limited, gender matching could not be achieved for the vasculitis patients with pulmonary vascular involvement in this study.

Previous studies [32, 33] have demonstrated that vasculitis involving the pulmonary vasculature can lead to vascular wall thickening, luminal stenosis or occlusion, resulting in a reduction of distal pulmonary vessels. The involvement of the pulmonary arteries can lead to severe pulmonary hypertension, resulting in structural remodeling of the distal pulmonary microvasculature and an increase in both pressure and resistance in the distal pulmonary venules [34–36]. Previous quantitative studies on IPVV have primarily focused on diseases such as COPD [15, 37], smoking-related changes [38], pulmonary hypertension [39], coronavirus disease 2019 (COVID-19) [14] and idiopathic pulmonary fibrosis (IPF) [40]. However, to date, no studies have been reported on the automatic segmentation of pulmonary vessels and measurement of IPVV in vasculitis patients with pulmonary vascular involvement. The results of this study demonstrated that the IPVVa in vasculitis patients with pulmonary vascular involvement was significantly lower than that in CTPA-negative subjects. Therefore, IPVVa may serve as a quantitative indicator for assessing pulmonary arterial involvement in patients with vasculitis affecting the pulmonary vasculature. Whether automatically measured IPVVv can indicate pulmonary venous involvement in vasculitis patients with pulmonary vascular involvement remains to be verified. Similarly, it remains to be determined whether TIPVV, which includes IPVVv, can serve as an indicator of overall pulmonary vascular involvement in these patients.

A previous study [22] has reported measurements of IPVV in patients with pulmonary hypertension using vessel diameter thresholds of < 0.8 mm, < 1.2 mm and < 1.6 mm; however, no studies have investigated IPVVa and IPVVv across different vessel diameter ranges in vasculitis patients with pulmonary vascular involvement. Our study first classified the pulmonary vessels into five diameter-based groups and applied a computer-aided pulmonary vascular segmentation algorithm to segment pulmonary arteries and veins. The results showed that the IPVVa in the 0.8–1.6 mm and 2.4–3.2 mm vessel diameter groups of vasculitis patients with pulmonary vascular involvement were lower than those of CTPA-negative subjects, and both differences were statistically significant. Therefore, the IPVVa in the 0.8–1.6 mm and 2.4–3.2 mm vessel diameter group can be used to quantitatively assess vasculitis patients with pulmonary vascular involvement, while differences in other diameter groups were not significant. According to previous literature [41], the segmentation of proximal pulmonary arteries is more affected by accompanying bronchi than that of distal pulmonary arteries. Additionally, the relatively small sample size in this study may have introduced some bias between the quantitative measurements and the actual conditions.

This study demonstrated that vasculitis patients with pulmonary vascular involvement exhibited higher IPVVv across all vessel diameter groups compared to CTPA-negative subjects. Notably, significant differences were observed in the 1.6–2.4 mm and 3.2–4.0 mm vessel diameter groups. This may be attributed to pulmonary hypertension-induced remodeling of the distal pulmonary microvasculature, which results in elevated pressure and resistance in the distal pulmonary veins. However, CTPA imaging primarily emphasizes the enhancement of the pulmonary arteries. Consequently, the visualization of pulmonary veins may be partially affected due to the scanning phase optimized for pulmonary arteries. In addition, only pulmonary arteries were evaluated by radiologists, while pulmonary veins were not assessed. Therefore, although an increase in IPVVv was observed in this study, this finding should be interpreted with caution and considered exploratory. Whether it accurately reflects the true condition of pulmonary veins in vasculitis patients with pulmonary vascular involvement remains to be determined. Future studies incorporating dedicated venous-phase imaging protocols will be necessary to validate these preliminary observations. In contrast, the IPVVa findings are more robust, as they are supported by arterial-phase optimization and direct radiological evaluation.

Pulmonary arterial involvement in patients with vasculitis affecting the pulmonary vasculature was quantitatively analyzed. In addition, this approach enables intuitive visualization of vascular involvement across different vessel groups. To the best of our knowledge, this segmentation algorithm has not been previously reported in the literature.

This study had some limitations. First, this was a retrospective analysis with a relatively small sample size. Age- and sex-matching between the vasculitis and CTPA-negative subjects was not performed. Given that age and sex may influence pulmonary vascular structure and function, future multicenter studies with larger, well-characterized cohorts are needed to enable age- and sex-matched analyses. Second, patients were not stratified according to the 2012 Chapel Hill Consensus Conference (CHCC) classification due to the limited cohort size. Most of our patients (179/207, 86.5%) were diagnosed with large-vessel vasculitis, while only 28 patients (13.5%) had medium- and small-vessel vasculitis. Given that different vasculitis entities affect vessels of different sizes, pooling these subtypes may obscure disease-specific patterns in IPVV. The small sample size of the medium- and small-vessel subgroup (*n* = 28) precluded meaningful stratified statistical analyses. Therefore, our findings are predominantly representative of large-vessel vasculitis. Future studies with larger, subtype-balanced cohorts are warranted to explore subtype-specific patterns. Third, lobar differentiation of IPVVa and IPVVv was not performed, so TIPVV may not fully reflect the extent of pulmonary vascular involvement. Fourth, this study employed a tube voltage of 120 kVp for CTPA acquisition and did not investigate the impact of varying tube voltages on IPVV measurement. Fifth, this study did not include completely healthy volunteers as controls. Although participants with known pulmonary diseases affecting pulmonary vasculature were excluded, the presence of subclinical or undiagnosed cardiopulmonary conditions cannot be entirely excluded. Sixth, the use of multiple comparisons in this study increases the chances of Type Ⅰ errors, which should be considered when evaluating the findings. We did not adjust for multiple comparisons, as this could lead to Type II errors [42]. Seventh, clinical correlation analyses were not performed in this study, which limits the direct clinical interpretation of IPVV and its potential utility in guiding patient management. Prospective studies integrating imaging and clinical data are needed to assess the potential clinical utility of IPVV as a biomarker in vasculitis. Finally, the resolution limits imposed by the CT pixel size present challenges in accurately quantifying the pulmonary vessel volumes, which may affect measurement precision.

## Conclusions

In summary, a computer-aided pulmonary vascular segmentation algorithm enables noninvasive analysis of the morphological characteristics of small pulmonary vessels, facilitating both 3D visualization and quantitative measurement of IPVV in vasculitis patients with pulmonary vascular involvement.

## Data Availability

The study data are available from the corresponding author on reasonable request.

## Abbreviations

BSA: Body Surface Area
COPD: Chronic Obstructive Pulmonary Disease
CTPA: Computed Tomography Pulmonary Angiography
HU: Hounsfield Units
IPVV: Intrapulmonary Vessel Volume
IPVVa: Intrapulmonary Arterial Vessel Volume
IPVVv: Intrapulmonary Venous Vessel Volume
TIPVV: Total Intrapulmonary Vessel Volume
3D: Three-Dimensional
%CSA: Cross-Sectional Area Percentage of Small Pulmonary Vessels
